# Rapid Nucleic Acid Diagnostic Technology for Pandemic Diseases

**DOI:** 10.3390/molecules29071527

**Published:** 2024-03-29

**Authors:** Yu Lei, Dawei Xu

**Affiliations:** 1 CAS Key Laboratory for Biomedical Effects of Nanomaterials and Nanosafety, National Center for Nanoscience and Technology (NCNST), Chinese Academy of Sciences (CAS), Beijing 100190, China; leiy2021@nanoctr.cn; 2 University of Chinese Academy of Sciences (UCAS), Beijing 100049, China

**Keywords:** rapid diagnostics, nucleic acid detection, COVID-19

## Abstract

The recent global pandemic of coronavirus disease 2019 (COVID-19) has enormously promoted the development of diagnostic technology. To control the spread of pandemic diseases and achieve rapid screening of the population, ensuring that patients receive timely treatment, rapid diagnosis has become the top priority in the development of clinical technology. This review article aims to summarize the current rapid nucleic acid diagnostic technologies applied to pandemic disease diagnosis, from rapid extraction and rapid amplification to rapid detection. We also discuss future prospects in the development of rapid nucleic acid diagnostic technologies.

## 1. Introduction

In recent years, with numerous pandemic diseases such as COVID-19 threatening countries worldwide [1], the need for a low-cost, high-sensitivity, and rapid diagnostic technology has been highlighted more than ever before. RT-qPCR technology, the gold standard within the diagnostic field [2], uses specific primers and high-performance enzymes to achieve nucleic acid amplification and disease diagnosis. The addition of fluorescent dyes or probes empowers RT-qPCR with real-time quantitative ability. However, the speed of current RT-qPCR technology cannot cope with rapid screening requirements in the face of a sudden worldwide pandemic disease outbreak. Nowadays, the top choice for rapid diagnostic technology is antibody IgG/IgM testing, which is fast and cheap. However, antibody-based diagnostic testing exhibits a high false positive rate and low sensitivity, which limits its potential application as a viable standard test during large-scale pandemic disease screening [3]. These dilemmas have led to urgent demand for the development of another reliable diagnostic technology that takes both speed and accuracy into account. Rapid diagnostic technology based on nucleic acid detection, which can achieve accurate clinical diagnosis and advances from sample processing to a result within 30 min, has thus become a major research focus.

**Figure 1:**
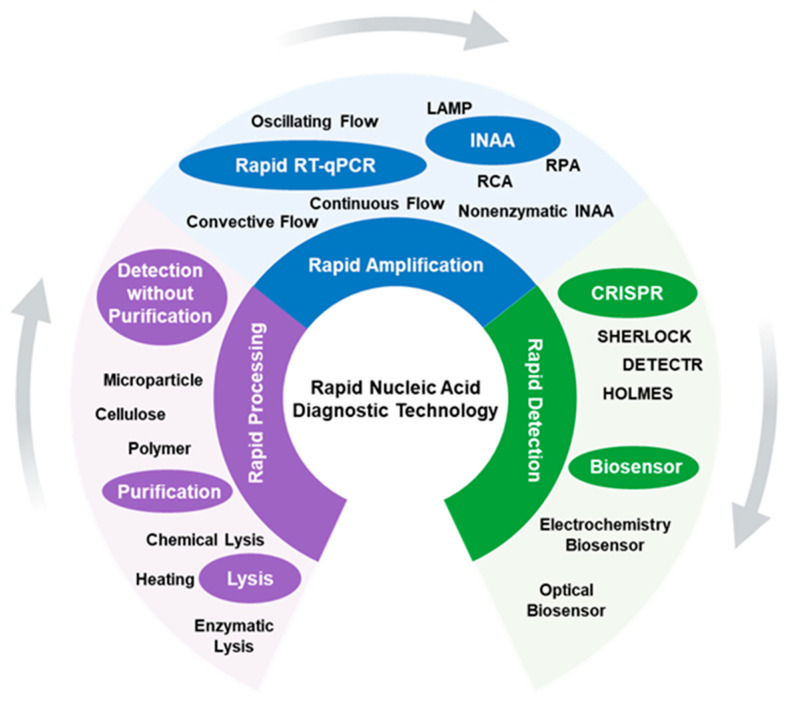
General development direction of rapid nucleic acid diagnostic technology.

At present, rapid diagnostic technologies are developing in a number of directions (Figure 1). The first aspect involved in rapid nucleic acid diagnostic technology is rapid nucleic acid extraction. Virus nucleic acid extraction mainly includes two steps: virus lysis (via chemical, heating, and enzymatic methods), and nucleic acid purification (magnetic beads-based, filter-based, and so on). For faster sample processing, extraction and purification-free detection technology has been reported by Visseaux et al. and Blumenfeld et al. [4,5]. According to different requirements for nucleic acid quality and purity, various rapid amplification technologies are compatible with different rapid extraction methods. For example, extraction-free detection is more suitable when paired with isothermal amplification (INAA), due to the fact that isothermal amplification is more tolerated by the amplification inhibitory fraction from the lysed sample [6]. After extraction, how to amplify the target nucleic acid rapidly and effectively is the most concerning issue at present. The traditional RT-qPCR improvements, including heat-conducting and thermal-cycling system improvements, have always been the focus, and remarkable progress has been obtained in these areas, such as via the deployment of convective thermocycling, oscillating flow, and continuous flow. In addition to traditional RT-qPCR improvements, several other rapid detection technologies have been developed, among which the most typical and widely studied representative is INAA. Without considering the time consumption associated with thermal cycling, the primary intended functions of INAA are to enhance amplification efficiency, improve sensitivity, and simplify the instrument. Apart from extraction and amplification, rapid detection technologies for amplification products, such as CRISPR and biosensors, have been developed as well. These new detection methods can monitor tiny amounts of biomarkers at the early stages of amplification, thereby reducing the time required for diagnostics. In some high-precision biosensors, nucleic acids extracted from viruses can even be detected without any amplification. 

**Figure 2:**
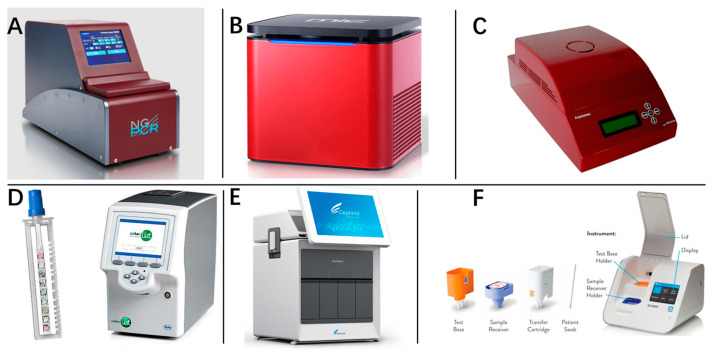
Rapid nucleic acid diagnostic machines. (**A**) NextGen PCR^®^; (**B**) Mic qPCR Cycler, (**C**) αAmp^®^ Cycler, (**D**) Cobas^®^ Liat System (**E**) GeneXpert^®^ System, (**F**) Abbott ID NOW™.

Based on current rapid diagnostic technologies, numerous commercial rapid nucleic acid diagnostic machines are in competition (Table 1, Figure 2), and this has triggered a new technological revolution in the field of clinical molecular diagnostics. While many of these instruments build upon enhancements to traditional RT-qPCR, notable examples include the Mic qPCR Cycler [7] and aAmp^®^ Cycler [8], which employ magnetic induction and infrared heating, respectively, to refine the heating element. Another significant advancement is represented by the NextGene-PCR [9], which uses an oscillating flow PCR system to transform the thermal cycling approach. Additionally, there has been a growing influx of instruments rooted in novel technological principles, such as ID Now COVID-19 [10], Lucira COVID-19 [11], and BG-Nova-X8 [12], all of which operate on the principles of isothermal amplification. The BG-Nova-X8 stands out as it integrates CRISPR technology, moving beyond traditional fluorescent probe detection. This diverse array of devices not only marks an advance in rapid diagnostic methods, but also indicates a shift towards more efficient and innovative solutions in molecular diagnostics.

In previous reviews, rapid diagnostic technology developments relating to pandemic diseases, and especially to COVID-19, have been elaborated on in several articles from different perspectives. Kostyusheva et al. [21] discussed the development of the CRISPR-Cas system for diagnosing infectious diseases and the obstacles hampering rapid translation of the CRISPR-Cas technology into practice. Lino et al. [22] summarized the biosensors used in clinical diagnosis and emphasized the analyte detection capability of biosensors in a reduced amount of time. Boehringer et al. [23] focused on lateral flow assays, which were one of the most used rapid technologies in infectious disease diagnosis. Despite the progress seen in the field in recent years, none of the abovementioned reviews focused on summarizing all of the current rapid diagnostic technologies based on nucleic acid detection. In this review, various widely used rapid diagnostic technologies are discussed, along with their merits and limitations. Comparisons between different technologies are provided. In the last section, the future development direction of rapid nucleic acid diagnostic technology is also prospected.

## 2. Rapid Processing

From the perspective of the overall standard testing process, the first consideration should be how to extract high-quality nucleic acids rapidly and efficiently from clinical samples. Clinical epidemic samples are usually collected in the form of nasopharyngeal (NP) swabs, oropharyngeal (OP) swabs, throat (TH) swabs, blood, serum, sputum, fecal matter, or urine [24]. In actual application, NP, OP, and TH swabs are the most recommended choices while facing the challenge of mass epidemic screening [25,26]. During the COVID-19 pandemic, medical supplies and labor requirements to facilitate rapid collection of clinical samples became scarce. Swabs, a low-cost, portable, non-invasive, self-directed method for sample collection, became mainstream and relieved many pressures for society. In addition, this self-collection method can greatly reduce the risk of infection for testers. Swabs should be considered the preferred mode for clinical sample collection. Additionally, in some point-of-care testing (POCT) devices, saliva, which does not require additional sampling tools and has stable sampling results, was also preferred in many studies and showed the same test effects as NP and TH swabs [27,28,29].

After collection, samples should be processed uniformly to extract nucleic acids from virus particles. The traditional sample processing strategy is based on commercial nucleic acid extraction kits and automatic nucleic acid extraction machines. However, these time-consuming commercial extraction methods are high-cost and need to be operated in environmentally controlled settings. Esbin et al. [30] reviewed the recent COVID-19 sample processing methods and showed that nowadays, sample processing has advanced into a fast, low-cost, and high-throughput processing stage with the availability of many new technologies and strategies. Sample processing strategy can be divided into two steps: lysis and purification. Typical lysis methods include chemical, heating, and enzymatic lysis strategies. In practice, testing institutions prefer chemical and heating lysis over enzymatic lysis, because enzymatic lysis requires incubation of clinical samples with protease in the buffer for a long period of time, which goes against the idea of rapid diagnosis. Additionally, the storage and cost of protease are also major problems affecting the viability of actual use. Smyrlaki et al. [31] tested different pyrolysis temperatures and times, and found that the best treatment condition was heating at 95 °C for 5 min. In this study, the pyrolyzed samples were directly used for RT-qPCR amplification, and comparable results to the purified sample were obtained. Direct amplification tests were also performed on clinical samples lysed with detergents such as Triton-X-100 and Tween-20. The results demonstrated that direct SARS-CoV-2 RT-PCR could be applied on a detergent-containing sample while a certain inhibitory effect for RT-qPCR still existed. Merindol [32] also tested the direct amplification effect of clinical samples of COVID-19 without purification and drew the same conclusion. Direct use of pyrolytic clinical samples is a time-saving and environment-friendly method which can be a great choice for rapid sample processing. However, these studies showed the potential problems of high-temperature cracking, which might lead to a certain extent of fragmentation for long fragment nucleic acids and thus influence the test results. The work of Lownik et al. [17] also showed the importance of nucleic acid purification under certain extreme amplification conditions.

**Figure 3:**
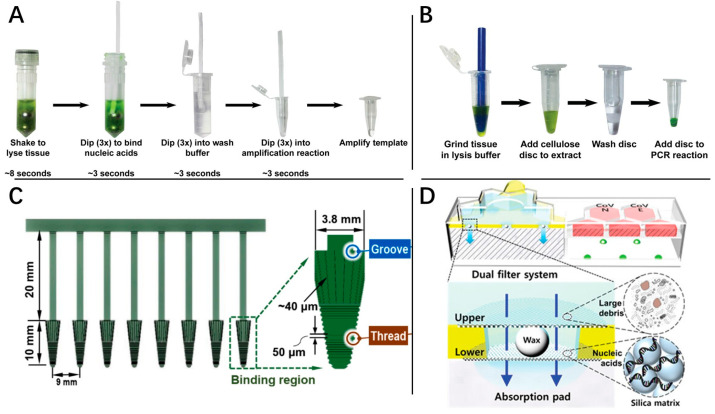
Rapid purification methods. (**A**) A rapid (30 s), equipment-free purification protocol of nucleic acids using easy-to-make cellulose dipsticks. (**B**) A cellulose disk to extract nucleic acids from plants, animals, and microbes in under 30 s. (**C**) Schematic diagram and microscopic images of the key structures of the 3D-printed separator applied in an 8-tube strip. (**D**) Dual filter system for ultrafast on-chip NA purification and enrichment without clogging. The upper membrane comprises large pores to filter out large debris, and the lower membrane is composed of small pores coated with silica particles to capture nucleic acids.

In rapid clinical diagnosis, there is often no additional heating equipment to inactivate clinical samples during the sample transportation process. Chemical lysis is a much more common strategy for large-scale screening. Considering that these biological samples and lysis buffer often have complex components that are not conducive for downstream detection work, such as nucleic acid digestion enzymes and a high concentration of guanidine salt, an effective nucleic acid purification method could play a vital role in diagnostics. For the purification process, various rapid nucleic acid purification methods were implemented and are shown in Figure 3. Mason [33] demonstrated a rapid (30 s), equipment-free purification protocol for nucleic acids using easy-to-make cellulose dipsticks (Figure 3A). Based on the generalized adsorption-cleaning-elution flowsheet of the filter-based nucleic acid purification method, this study used a cellulose dipstick to replace the commonly used microcolumn and obtained a good purification effect. Following on from this, Mason et al. [34] designed a cellulose disk to extract nucleic acids from plants, animals, and microbes in under 30 s (Figure 3B). Additionally, Qian et al. [35] and Li et al. [36] also adopted filter and polyacrylic acid (PAA), respectively, to fabricate rapid purification dipsticks. Qian’s work achieved a 2 min rapid nucleic acid purification with CRISPR/Cas12a-mediated isothermal amplification for visual detection of African swine fever virus, with a detection limit of 1 copies/μL. Li et al. integrated 3D printing techniques for high throughput and convenient DNA separation (Figure 3C). In addition to dipstick and disk forms, many studies have also designed different partial integration devices to shorten the purification time. Song et al. [36,37] demonstrated an RT-LAMP chip that can detect clinical samples within 30 min through preconcentration with a limit of detection (LOD) of 0.5 copies/µL (Figure 3D). Nucleic acids were adsorbed onto the filter membrane with silica particles and then directly infiltrated with LAMP reaction solution, and thus the elution and 500-fold pre-concentration of nucleic acids could be achieved.

For all the studies mentioned above, the sample collection strategy was similar: clinical samples were collected by swabs, and then chemically or physically lysed. In contrast, how to process the cleaved mixture is the main focus in rapid sample processing. Most research is devoted to developing a high-speed, easy-to-operate, and efficient purification method, while others offer the answer of direct testing the crude lysates without purification. At present, the dilemma of the former is how to reduce the operation time on the premise of reducing the associated loss of nucleic acid. Many rapid nucleic acid purification methods are unable to deal with low copy number samples well. The problem with the latter study type is that the stability of diagnostics cannot be guaranteed due to the diversity of clinical samples. For example, the proportion of sputum in patients with COVID-19 at different stages is dissimilar. In the face of these problems, micro and nanotechnology may provide the answer. Droplet technology, represented by digital PCR, can separate inhibitory components from nucleic acids without purification. This is possible because the droplet size is extremely tiny, and can only accommodate a single nucleic acid molecule. In some studies [38,39], hydrogels with nanopore structures were shown to achieve the same function. Sample miniaturization, to directly physically isolate inhibitory components, is a sample processing strategy that takes into account current mainstream research ideas. With the development of micro- and nano-processing technology, these may become the mainstream vehicles for rapid sample processing.

## 3. Rapid Amplification

### 3.1. Rapid RT-qPCR

For traditional RT-qPCR, the total amplification time can be more than one hour. Nowadays, with the exploration of RT-qPCR enzyme reaction kinetics, the time required for RT-qPCR is mainly affected by the instruments used, rather than the biochemical properties of the RT-qPCR reaction system [40,41,42]. After RNA reverse transcription, the PCR mainly consists of three stages: denaturation, annealing, and extension. In most cases, the annealing and extension stages can be carried out simultaneously. In research surrounding PCR kinetics [43], under ideal conditions, no hold time was required for denaturation and annealing while extension could occur during the transition between temperatures. Especially for short DNA amplicons, the holding time of these three processes could be sufficiently omitted [44]. For example, Millington’s work [41] showed that 98% of the PCR product could be denatured for 60 bp DNA amplicon under a denaturation time of 200 ms. Based on these theories, a multitude of creative instruments have been produced and can accomplish RT-qPCR within 30 min, which is known as rapid RT-qPCR. Developed by Wittwer et al. [45], the extreme PCR can accomplish clinical sample detection in 10 min. These rapid RT-qPCR systems were developed based on one of two main directions: reaction volume miniaturization, such as in capillary RT-qPCR, and reaction thermal cycling acceleration, such as in convective RT-qPCR. These new technologies emphasize the improvement of the instrument; in contrast, the past several decades of improvements in the field were focused on the reaction system, the enzyme performance, and the primer, as well as probe design.

Miniaturization of RT-qPCR reaction volume can dramatically improve its performance. The use of a combination of glass capillary tubes and airflow systems in place of traditional polypropylene tubes and solid-state heating modules was reported in [12]. The earliest application of a glass capillary/airflow system was in 1991 [46], and in terms of a real-time PCR, the earliest application was seen in the Roche LightCycler^®^ instrument in 1996 [47,48]. Compared to polypropylene tubes, glass capillary tubes have higher thermal conductivity and smaller sample requirements. The elongated container in the shape of capillary tube also demonstrates a higher surface-to-volume ratio. Some studies have further shown that the small volumes of glass capillary tubes could increase amplification efficiency and accelerate PCR [49,50]. Microfluidics is another technology suitable for meeting the thermodynamic performance requirements of rapid RT-qPCR. The micro/nano-level sample volume and high thermal conductivity substrate make microfluidic chips an excellent choice for rapid diagnosis [51,52]. Microfluidic chips can also easily achieve high integration of every clinical diagnostic technology, from virus lysis and nucleic acid extraction to RT-qPCR amplification and results visual output. Combined with the droplet technique [53,54], bulk samples can be further divided into numerous droplets to achieve multiplex detection. Ji et al. [55] launched a machine for automated multiplex nucleic acid tests of SARS-CoV-2 and influenza A and B infections. With high heat cycle performance and small reaction volume, both capillary and microfluidic chips can achieve rapid detection. However, for low copy number samples, and especially for clinical pandemic samples, a smaller volume within the reaction system is not considered better. Dong et al. [56] showed that when reaction volume is smaller than 1.3 μL, the PCR system is not suitable for clinical pandemic diagnostics due to limited nucleic acid templates.

**Figure 4:**
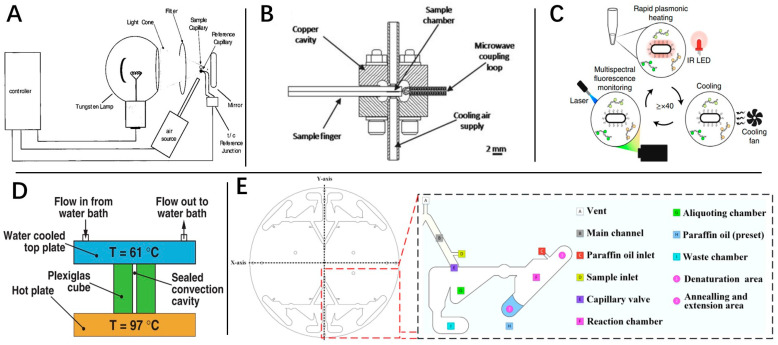
Rapid RT-qPCR studies. (**A**) Instrumentation setup for IR-mediated thermocycling in capillaries. (**B**) Schematic of re-entrant cylindrical microwave cavity. (**C**) Schematic of multiplexed real-time plasmonic RT-PCR, with heating driven by IR LEDs acting on AuNRs and cooling aided by a 12 V fan. A 488 nm laser and spectrometer setup provides real-time fluorescence detection and takes a measurement at the end of each annealing/extension hold. (**D**) Schematic of dual-end heating mode of convective thermocycling. (**E**) Diagram of the different parts of the oscillating chip and their function.

An engineered thermal-cycling system can also be a great boost for rapid RT-qPCR. The traditional thermal-cycling system worked by repeatedly heating and cooling the metal module loading the sample tubes to achieve a RT-qPCR thermal cycle. Traditional RT-qPCR machines, such as the Applied Biosystems 7500 Real-Time PCR System (Agilent Technologies Inc., Santa Clara, CA, USA), use a common semiconductor heating module and can achieve a 5.5 °C/s heating and cooling speed, according to the product manual. For one cycle from 60 °C to 95 °C to 60 °C, it takes almost 13 s, without considering holding time. In contrast, many new devices with direct heating methods have faster temperature changes. Infrared heating [57], microwave heating, and nanoparticles are the three most widely used non-contact heating methods [40,58] that can skip the heat transfer process to achieve rapid thermal cycles. Infrared heating and microwave heating are, respectively, based on the energy absorption from infrared rays and microwaves by liquid. Hühmer et al. [59] used infrared ray to heat a thin capillary tube of nanoliter reaction volume to achieve effective PCR in 30 min (Figure 4A). Although this work focused on the PCR process, the system showed the potential for rapid RT-qPCR. Similarly to infrared heating, microwave heating can also accomplish super-quick direct heating of reaction buffer (Figure 4B) [60,61]. Sun et al. [62] showed that microwave irradiation at a constant temperature could lead to faster nucleic acid strand exchange, but the potential of nucleic acid breakage upsurged, especially for long fragments. The nucleic acid breakage caused by microwave heating is still a considerable negative factor associated with this method. Nanoparticles are another technology implicated in rapid RT-qPCR that have generated great research interest. With the plasmon oscillation effect of metal nanoparticles, a direct heating method without the heating transformation process can be accomplished [63]. Nicole R. Blumenfeld et al. [5] demonstrated a plasmonic thermocycling system in which rapid thermal cycling was achieved via infrared excitation of gold nanorods (Figure 4C). The instrument showed a sample-to-result time of 22–23 min, including sample preparation, and a LOD of 2.2–4.4 copies/mL.

Innovative design of the thermal cycling mode is proposed for a rapid RT-qPCR system. Convective thermocycling is a novel thermocycling mode designed to increase the amplification speed and simplify the thermal cycling system. The convective PCR concept was first demonstrated in a cylindrical cavity by Krishnan et al. in 2002 (Figure 4D) [64]. The researchers used a single-end or dual-end heating source to create temperature gradients by natural convection, induced by density changes of fluids [65,66,67,68,69]. Although the convective RT-qPCR machine could be hard to design given the high requirements surrounding micro-hydrodynamics and thermodynamics, its conceptual simplification of the instrument design still demonstrated an enormous potential for POCT devices. In the following studies, Qiu et al. [70] adapted a thermal waveguide for precise temperature control of convective thermocycling. Khodakov et al. [71] fabricated a portable and battery-powered PCR assay that allowed for rapid and sensitive quantification of multiple DNA targets with single-nucleotide discrimination, which further advanced the application of convective RT-qPCR in terms of sensitivity and specificity. For temperature control of thermal cycling, oscillating and continuous flow systems have been proposed. The oscillating flow system successfully omits the temperature change of the heating element by moving reaction tubes back and forth between different preheated thermostatic heat sources [72]. Witter and Jared [73] were the first to propose the concept. They showed an extreme PCR system that delivered results in 15–60 s. An extreme RT-PCR, which delivered results in 2 min, was further shown in 2020 by Witter et al. [74]. In the follow-up study, Chen et al. [75] fabricated a 100-mm-long oscillating thermocycler and studied its specific performance in detail. Arumugam et al. [76] also demonstrated a specially designed thin-walled PCR tube and a water bath setup for oscillating flow RT-PCR, which could achieve RT-PCR in 12 min for clinical samples without an RNA extraction step. Corresponding to oscillating flow, continuous flow directly pushes reaction solution through a channel at different temperature intervals to achieve rapid thermal cycling [77]. Yang et al. [78] proposed a multiplex circular array-shaped continuous flow PCR microfluidic chip for on-site detection of bacteria, which successfully achieved PCR amplifying of 12 different target genes simultaneously within 23 min (Figure 4E).

As can be seen from the above studies, theoretical research on RT-qPCR has become very mature. Nowadays, more attention is being paid to the optimization of engineering. The emergence of convective thermocycling as a representative technology proves that RT-qPCR still presents unlimited possibilities, and is expected to continue to maintain its status as the mainstream method for molecular diagnostics.

### 3.2. Rapid Isothermal Amplification

Current limitations of rapid RT-qPCR center on thermocycling speed and amplification efficiency. The advent of INAA technologies provides researchers with a new approach to achieve rapid amplification without thermocycling. Based on the unique strand replacement activity of DNA polymerase, INAA can achieve nucleic acid amplification in a constant medium-high reaction temperature (25–65 °C) with designed primers. Without the demand for thermocycling, INAA is faster, of higher quality, and has lower instrument requirements [79]. Additionally, INNA is more tolerant of direct detection without nucleic acid purification, as compared to RT-qPCR. Lalli et al. [6] have demonstrated an extraction-free detection of SARS-CoV-2 from saliva with colorimetric LAMP, which enabled rapid and sensitive detection of <10^2^ viral genomes per reaction in 30 min. This characterization further accelerates and simplifies the INNA diagnostic process. At present, INAA technologies that have been extensively studied include loop-mediated isothermal amplification (LAMP) [80], rolling circle amplification (RCA) [81], recombinase polymerase amplification (RPA) [82], strand displacement amplification (SDA) [83], nucleic acid sequence-based amplification (NASBA) [84], and others. Among all of these INAA technologies, the amplification time varies from as low as several minutes to 30 min or more, and the diagnostic result for clinical samples is consistent with traditional RT-qPCR.

#### 3.2.1. Loop-Mediated Isothermal Amplification

**Figure 5:**
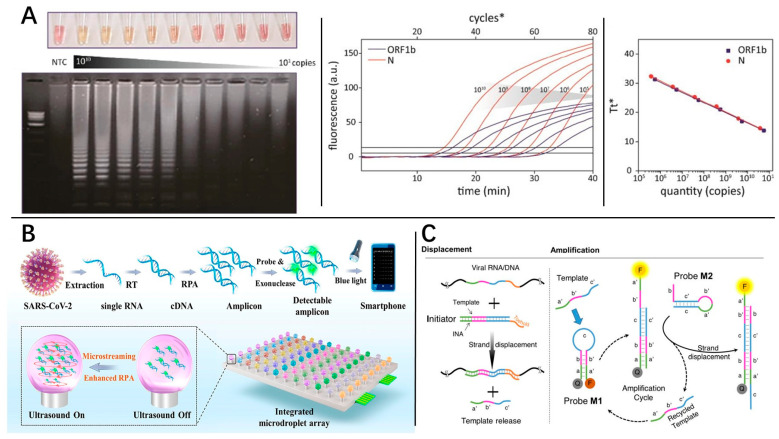
Different INAA technologies. (**A**) The color and gel electrophoresis results of colorimetric RT-LAMP with different copy numbers, as well as the associated amplification curve. (**B**) Schematic of the RPA reaction-based workflow of the integrated microdroplet array platform for ultrafast and high-throughput diagnosis of SARS-CoV-2. (Cycles*: fluorescence signals were collected at 30-s intervals and recorded as one cycler. T_t_*: represents the time at which the fluorescence was equal to the threshold value during LAMP cycles.) (**C**) Schematic diagram of TMSD technology. (The M1 and M2 probe need to be designed according to the three consecutive segments in the template, namely the a, a′, b, b′ and c, c′ sequences annotated in the figure).

LAMP was first described in 2000 by Notomi et al. [85] and has become one of the most used INAA technologies. With the help of four to six primers to identify six to eight different regions of the target DNA, the transcribed DNA can form a dumbbell-shaped or stem-loop DNA structure, which then initiates the subsequent amplification reaction. Klein et al. [86] compared the performance of RT-qPCR and RT-LAMP for SARS-CoV-2 diagnostics, and the results showed that RT-LAMP was faster and cheaper compared to RT-qPCR. Additionally, RT-LAMP results could be visualized through magnesium pyrophosphate precipitation during the reaction and could reduce the need for fluorescence detection instruments (Figure 5A). However, as reported by Dao et al. [87], colorimetric RT-LAMP detection of SARS-CoV-2 exhibited decreased sensitivity compared to RT-qPCR, which was mainly caused by the high false positive rate observed while using low copy number samples. RT-LAMP can be deployed as a multiplex diagnostic platform [88]. Kim et al. [89] demonstrated a multiplex RT-LAMP assay that could target open reading frame (ORF1b) and nucleocapsid (N) genes of COVID-19. The detection limit of the LAMP assay was 10^4^–10^5^ copies within 20–25 min.

#### 3.2.2. Rolling Circle Amplification

RCA was first developed by Fire, Andrew, and Si-Qun Xu in 1995 [90]. Unlike LAMP, the samples targeted by the RCA technique tend to be circular. For linear targets, a designed padlock probe is needed for amplification reactions. The reaction temperature of RCA is usually 37–42 °C. RCA products can be monitored in various forms, including gel electrophoresis [91], fluorescence [92], and the naked eye [93]. RCA is often used to detect microRNA and infectious pathogens. Reported by Khoothiam et al. [94], a multiple primer-mediated rolling circle amplification was developed to detect lung cancer-associated miRNAs. Liu et al. [95] also used the DNAzyme-based LRCA to detect miRNA-21. The signal output was based on a zinc (II) protoporphyrin IX (ZnPPIX) system, and the LOD was 1.019 fM. Qing et al. [96] also fabricated an electrochemical biosensor based on RCA and CRISPR/Cas12a technologies, and the calculated LODs for miRNA-21, Parvovirus B19 DNA, and adenosine-5′-etriphosphate were 0.83 aM, 0.52 aM, and 0.46 pM, respectively.

#### 3.2.3. Recombinase Polymerase Amplification

As one of the most used INAA technologies, RPA requires minimal sample preparation and offers excellent sensibility. RPA was first introduced in 2006 [97], and uses recombinase to aid primer binding to double-stranded DNA, thereby replacing the denaturation step at 95 °C. Compared to other INAA technologies, the advantage of RPA is its ability to generate amplicons up to 1 kb. Zhou et al. [82] demonstrated an RPA microdroplet array platform (Figure 5B) that integrated a mini-pillar array and ultrasonic components to conduct microstreaming. In the presence of microstreaming, the amplification time was reduced to 6–12 min, which was 38.8–59.3% shorter than the control without microstreaming, and the LOD was 0.42 copies/μL for SARS-CoV-2. A winning combination of microfluidics and RPA has been shown in this work. 

#### 3.2.4. Nonenzymatic INAA

Toehold-mediated strand displacement (TMSD) is an alternative cost-effective isothermal amplification technique based on competitive hybridization reactions (Figure 5C) [98,99]. Unlike other INAA technologies, interestingly, TMSD is a rare nonenzymatic amplification technology. Mohammadniaei et al. [98] demonstrated an assay based on TMSD, termed non-enzymatic isothermal strand displacement and amplification (NISDA), which was able to quantify 10 RNA copies/µL in 30 min without reverse transcription. In the testing of 164 COVID-19 oropharyngeal clinical samples, the NISDA assay was 100% specific and 96.77% sensitive in the laboratory setting.

As the most considered novel molecular diagnosis method in recent years, INAA has been developed rapidly. However, at present, existing theoretical research on isothermal amplification technology is still not deep enough, and the overall technical standards are difficult to unify due to the diversity of detection strategies. It will be important in future to consider how to conduct systematic sorting and in-depth theoretical research on all of the isothermal amplification technologies and to determine how to refine their market standards accordingly.

From all that is mentioned above, it is clear that both rapid RT-qPCR and rapid isothermal amplification achieve amplification of low copy number samples using different strategies. Generally, RT-qPCR is more stable, and isothermal amplification is faster. At present, the mainstream status of RT-qPCR technology remains the gold standard, but with the ongoing development of technology, isothermal amplification technology has exhibited more possibilities. Learning from experience with, and the theoretical basis of, RT-qPCR research, more in-depth research on isothermal amplification technology will become the main development trend in the future.

## 4. Rapid Detection

The traditional detection method for amplified nucleic acids is based on fluorescence dyes or fluorescence probes. While fluorescence probes have higher sensitivity as well as specificity and are more widely recognized in diagnosis, fluorescence dyes are cheaper and there is no need for a separate design for each target. However, both fluorescence dyes and probes need sophisticated fluorescence detection systems, along with amplification before detection when facing low copy number samples. For INNA, to simplify the instrument, most studies used the macroscopic color change of the tested solution to characterize amplification results. However, this semi-quantitative method was not sensitive enough and could only be applied to a single target [100]. In recent years, with the development of CRISPR technology, researchers have adapted the CRISPR/Cas system to characterize INAA results and have made great breakthroughs. In addition, biosensors are also evolving with the development of material characterization and micro/nano-fabrication technologies. 

### 4.1. CRISPR

**Figure 6:**
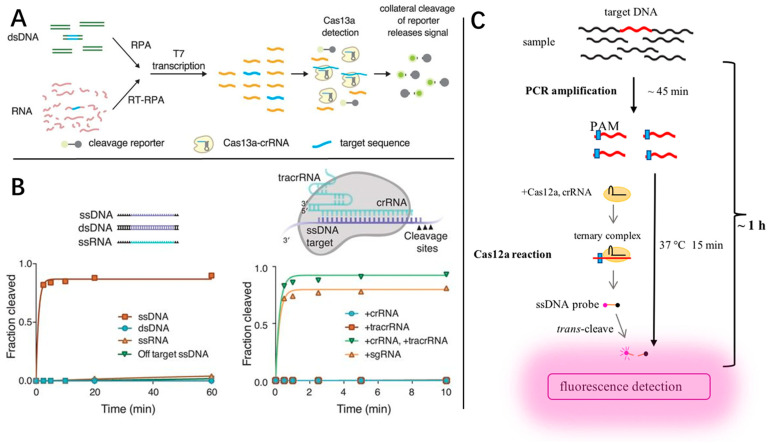
The schematic detection mechanism of (**A**) Specific High-Sensitivity Enzymatic Reporter UnLOKing (SHERLOCK), (**B**) DNA endonuclease-targeted CRISPR trans reporter (DETECTR) and (**C**) a one-hour low-cost multipurpose highly efficient system (HOLMES).

The CRISPR/Cas9 system is widely known as a gene-editing tool for its efficient and specific cleavage of nucleic acids. Along with the mining of its other novel properties, the CRISPR/Cas system has been gradually used as a rapid and ultra-sensitive diagnostic method based on the products amplified by RT-PCR or INAA [101]. Reported by Zhang et al., the CRISPR-Cas13 molecular detection technique has been developed and is known as Specific High-Sensitivity Enzymatic Reporter UnLOKing (SHERLOCK) (Figure 6A) [102]. Unlike the selective cleavage of Cas9, Cas13 has collateral cleavage characteristics and can cleave all substrates in the reaction system [103]. SHERLOCK is based on the amplification products of RPA or RT-RPA. Once Cas13 identifies the target sequence, the fluorescence group is cleaved from the quenching group and generates a fluorescence signal. The LOD of SHERLOCK can be at the level of attomoles. In the follow-up study [104], the lateral flow technology for visual readout was further added to replace the need for fluorescence detection. Although instrument-free testing was achieved, the total detection time for clinical samples was increased to 90 min. With a fluorescence detector, the detection time could be reduced to less than 23 min. Apart from SHERLOCK, more proteins from the Cas family were adapted for rapid diagnosis. Doudna et al. [105,106] developed DNA endonuclease-targeted CRISPR trans reporter (DETECTR) technology based on the RPA-Cas12a system and Cas14 (Figure 6B). The major difference between these two technologies is the adaptation of different reporter molecules. Li et al. [107] combined Cas12a and traditional PCR technology and developed a one-hour low-cost multipurpose highly efficient system (HOLMES) (Figure 6C). 

In these works, the CRISPR/Cas diagnosis system showed its exceptional potential for increasing LOD, simplifying instrumentation, low diagnostics cost, and a great potential to be the next generation of diagnosis [108] compared to the traditional fluorescence methods. However, various challenges such as quantitative detection limitation [109], off-target effects [110], and target sequence limitations [111] are still in the way and need to be overcome in future studies.

### 4.2. Physical and Chemical Biosensor

Both traditional and CRISPR detection methods depend on nucleic acid amplification to detect trace amounts of biomarkers contained in clinical samples. Based on electrochemistry [112,113,114,115,116], optics [117,118,119], and other principles, biosensors can convert the biological response into a visual physical or chemical signal to accomplish amplification-free and enzyme-free detection.

#### 4.2.1. Electrochemistry Biosensor

**Figure 7:**
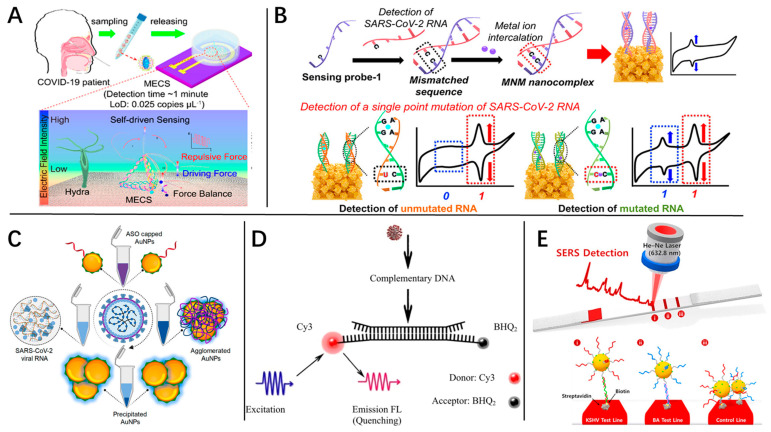
Different types of biosensors. (**A**) Schematic diagram of MECS for ultrasensitive detection of SARS-CoV-2 RNA by automatically controlling the position of the electrochemical indicator. (**B**) Electrochemical nanobiosensing system for detecting SARS-CoV-2 RNA and determining a single point mutation. The signal of metal ions was measured by cyclic voltammetry and the red/blue boxes marked the signal of the conserved/ mutant regions of SARS-CoV-2, respectively. (**C**) Schematic representation for the selective naked-eye detection of SARS-CoV-2 RNA mediated by the suitably designed ASO-capped AuNPs. (**D**) Schematic of the Cy3-based biosensor for the detection of complementary DNA. (**E**) The schematic diagram of SERS detection of SARS-CoV-2 based on LFIA.

The electrochemistry principle is a commonly used technology in biosensor design. The electrochemical nucleic acid biosensor is based on electrochemical signal changes during nucleic acid hybridization with single-base specific recognition and high sensitivity. Initially, the passive hybridization adopted in most DNA-based electrochemical biosensors required several hours to reach a detectable signal level [112], which bottlenecked the rapid detection capability. An external electric field was then demonstrated to accelerate the hybridization interaction. Edman et al. [120] were the first to reveal the synergy between nucleic acid interaction and external electric fields. The follow-up works [121,122] demonstrated a dramatic reduction in reaction time under a non-sinusoidal alternating voltage. With the development of two-dimensional materials including graphene and graphene-related materials, transition metal carbides, carbonitrides, nitrides (MXenes), and transition metal dichalcogenides (TMDs) [123], extremely high sensitivity, to the degree of almost a single nucleotide, and a rapid rate of response has been shown. For example, Ji et al. [124] demonstrated a graphene microelectrode biosensor, modified with a tentacle and a trunk, which could detect SARS-CoV-2 RNAs within 1 min down to 4 copies in 80 μL without nucleic acid amplification (Figure 7A). In recent years, with the development of electrochemistry, many other technologies have been used to improve the sensitivity and diagnostic speed of biosensors. The addition of redox metal ions is another effective strategy to enhance detection speed and sensitivity. Specific redox metal ions can selectively and effectively intercalate into double-stranded nucleic acids to stabilize construction when sequence mismatch occurs [125,126]. Based on this phenomenon, Yoon et al. [127] designed a sensing probe that could hybridize with target RNA to numbers of mismatched sequences and thus directly used the intercalated redox metal ions as a redox probe (Figure 7B). Due to intercalated metal ions having outstanding redox properties, a higher sensitivity could be achieved compared to traditional probes. Additionally, based on an entropy-driven amplified electrochemiluminescence (ECL) strategy, Fan et al. [128] modified DNA tetrahedrons on the surface of the electrode to enhance the ECL intensity, and achieved high sensitivity for SARS-CoV-2 with a LOD down to 2.67 fM.

#### 4.2.2. Optical Biosensor

Optical biosensors are another technology that have gained widespread adoption. These technologies include colorimetric biosensors, fluorescence biosensors, surface-enhanced Raman scattering (SERS) biosensors, and Surface Plasmon Resonance (SPR) biosensors. Optical biosensors convert the optical signals of probes or biomarkers to a real-time qualitative or quantitative result.

A colorimetric biosensor is a traditional optical biosensor that records detection results through the color change of the solution. Moitra et al. [129] reported a colorimetric biosensor based on gold nanoparticles (AuNPs) (Figure 7C), which could detect the N-gene of positive COVID-19 cases within 10 min from isolated RNA samples, with a LOD of 0.18 ng/μL of RNA. The advantages and disadvantages of colorimetric biosensors are obvious: they pose an extremely simple detection method, but have low sensitivity. Compared to other optical biosensors, colorimetric biosensors are more compatible with immune-based detection methods from the point of view of sensitivity.

The fluorescence biosensor is the most used optical biosensor. It adopts fluorescence materials [130,131] to perform signal output. The classic fluorescence biosensor is based on fluorescence resonance energy transfer (FRET), which was first proposed by Föster in 1948 [132]. For pandemic disease diagnosis, the target nucleic acid sequences competitively match to the acceptor molecules, changing the distance between the donor and receptor molecules, which results in fluorescence quenching or activation. Based on FRET, Bardajee et al. [133] used Cy3 as a donor pair, and BHQ2 as a receptor pair for the detection of clinical COVID-19 RNA samples (Figure 7D).

Raman spectroscopy is a widely used optical characterization method for molecular vibrations. With the help of SERS [134], the weak signal of Raman spectroscopy was further enhanced, and the SERS biosensor was developed. SERS biosensors can be divided into labeled [135,136,137] and label-free SERS sensors [138,139,140]. For labeled SERS, a high Raman scattering cross-section reporter molecule is used, and the reporter molecule can interact with the target molecules on the biosensor. While detecting, the reporter molecule affects the quantitative and qualitative results of the target molecule. On the contrary, label-free SERS directly detects the Raman spectroscopy signal of target molecules that are captured by the substrate. In comparison, the label-free SERS biosensor can show more information on target molecules, but faces the challenge of a weak spectroscopy signal. The labeled SERS biosensor is more commonly used, but is complicated to design. Wang et al. [141] demonstrated a new class of surface-enhanced Raman scattering (SERS)-based lateral flow assay (LFA) biosensor (Figure 7E), which could detect Kaposi’s sarcoma-associated herpesvirus (KSHV) and bacillary angiomatosis (BA) DNA in 20 min.

SPR is a traditional optical phenomenon [117]. While the light is completely reflected on the surface of prism or metal film, a vanishing wave will form and go into the photohydrophobic medium. In a particular situation, the vanishing wave can resonate with the plasma wave of the medium itself, and this resonance phenomenon is called SPR. Based on this phenomenon, the SPR biosensor records the change in resonance angle to achieve a real-time absorption and dissociation detection of a target molecule on the detector surface [142]. SPR biosensors could be divided based on their different detection methods into intensity-modulated SPR biosensors, angle-modulated SPR biosensors, wavelength-modulated SPR biosensors, and phase-modulated SPR biosensors. For SPR technology, the most outstanding advantages are high sensitivity and irreplaceable dynamic detection [143]. Akib et al. [144] demonstrated the development of an Au/PtSe2/graphene-coated SPR sensor. Based on different ligand analytes, COVID-19 viral spike RBD, single-standard RNA, and anti-spike protein (IgM, IgG) were successfully detected.

With regards to physical and chemical biosensors, most of the associated problems are related to low detection stability and high costs. In contrast with the traditional fluorescent dye detection method, biosensors often need to be designed separately for each target. The design cost and preparation time greatly reduce the application value of biosensors. In addition, the high sensitivity of biosensors also mean that they are more susceptible to environmental interference, which affects detection stability. At present, most biosensors are still at the level of theoretical and laboratory research. In the future, more research must focus on the solutions to engineering problems and the promotion of practical application.

In recent years, more and more studies have begun to focus on the simplification of molecular diagnostic equipment, such as POCT. In this process, rapid detection technology plays a critically important role. On one hand, the strategy of rapid detection technologies is to reduce the time required for nucleic acid amplification by improving the sensitivity of nucleic acids, resulting in less need for front-end sample processing and nucleic acid amplification. In addition, many new technologies, such as CRISPR and biosensors, are also trying to use novel detection methods to replace the traditional fluorescence detection methods, such as the colorimetric and electrochemical detection methods. By using these new detection methods, the demand for complex fluorescence detection equipment can be further reduced. Although the research into rapid detection technology still faces many bottlenecks in practical application, the subversive changes in molecular diagnostic devices brought about by rapid detection technology cannot be ignored. At present, few rapid detection strategies can significantly reduce the speed of molecular diagnosis in practical application. Most of the so-called rapid diagnostic technologies are another form of colloidal gold test strip. Rapid detection technology is the key to ensuring high sensitivity and accuracy in nucleic acid detection strategy, while at the same time being comparable to immunochromatography in terms of cost, convenience, and diagnosis time.

## 5. Conclusions and Prospects

According to the World Health Organization (WHO), the ideal pathogen diagnostic system should be low-cost, sensitive, accurate, rapid, and require little or no specialized instrument or technical assistance [145]. During COVID-19 epidemics, nucleic acid diagnostic technologies, with RT-qPCR or INAA at the core, were the most used commercially available diagnostic strategies. Antibody IgG/IgM testing dipsticks were also used as an auxiliary means of self-testing. Those two diagnostic strategies passed the test of the epidemic. However, with extended testing time and high dependence on professional testing laboratories, the current commercially available diagnostic systems are still under WHO criteria. 

During the COVID-19 epidemic, numerous aforementioned rapid diagnostic technologies emerged, but were still in the early development stage. Most of the emerging rapid diagnostic technologies are still at the laboratory level and are not suitable for clinical practice. For example, the dipstick-based rapid purification method showed promising results in laboratory settings, but was unable to process samples on a large scale and had potential risks of cross-contamination [33]. The CRISPR/Cas diagnostic system faced the problem of stability during transportation. Additionally, technology has its inherent limitations and cannot always achieve a broad spectrum of disease diagnosis. The most typical example is seen in extreme RT-qPCR technology. The shortened cycle time makes extreme RT-qPCR suitable only for high-quality nucleic acid samples and short amplicon amplification strategies [146]. The inherent high primer and enzyme concentration requirement of these reaction systems also leads to increased false positive reports when the primer specificity is not high enough. Nowadays, rapid diagnostic technologies have been developed across different aspects, including rapid extraction, rapid amplification, and rapid detection. In the future, integration of current rapid diagnostic technologies should be the prime direction.

## Figures and Tables

**Table 1:** Marketed rapid nucleic acid diagnostic machines for pandemic diseases.

Machine Name	Fundamental Principle	Detection Time	LOD	Company	Reference
Mic qPCR Cycler	Magnetic induction of a more lightweight thermal block	25 min	-	Bio Molecular Systems (Queensland, Australia)	[13]
αAmp^®^ Cycler	Infrared light directly heating, and airflow cooling	20 min	-	AlphaHelix Technologies AB (Stockholms Lan, Sweden)	[12]
NextGene-PCR	Pushing customized PCR plates with thin walls to different pre-heated temperature blocks	27 min	1000 copies/mL	Molecular Biology System B. V. (Goose, Zeeland)	[9]
GeneXpert^®^ System	Covering the reaction liquid using thin foils to facilitate efficient heat transfer	45 min	100 copies/mL	Cepheid Inc (Sunnyvale, CA, USA)	[14]
Cobas^®^ Liat System	Pressing the reaction sample forth and back with two differently pre-heated stamps	20 min	100–200 copies/mL	Roche Diagnostic International AG (Basel, Switzerland)	[15,16]
Light Cycler	Using thin capillary tubes	20 min	2000 copies/mL		[17]
ID Now COVID-19	LAMP	5 min (positive) 13 min (negative)	20,000 cpoies/mL	Abbott (Chicago, IL, USA)	[18]
BG-Nova-X8	Compatible with RAP CRISPR and magnetic bead extraction	30 min	-	Biogerm (Shanghai, China)	[12]
Alere i and q	Nicking enzyme amplification reaction	15 min	-	Alere (Newport Beach, CA, USA)	[19]
Lucira COVID-19	LAMP	11 min (positive) 30 min (negative)	-	Lucira Health (Emeryville, CA, USA)	[11]
Visby Medical COVID-19	Continuous flow system for PCR	30 min	500 copies/mL	Visby Medical (San Jose, CA, USA)	[20]

## Data Availability

No new data were created.
